# Predictors of Dropout from Inpatient Opioid Detoxification with Buprenorphine: A Chart Review

**DOI:** 10.1155/2014/965267

**Published:** 2014-10-30

**Authors:** Anders Hakansson, Emma Hallén

**Affiliations:** ^1^Department of Clinical Sciences Lund, Division of Psychiatry, Lund University, Baravägen 1, 221 85 Lund, Sweden; ^2^Malmö Addiction Center, SUS Malmö, Jan Waldenströms Gata 77, 205 02 Malmö, Sweden; ^3^Primary Care Unit, Kumla, Örebro County, Norra Kungsvägen 10, 692 31 Kumla, Sweden

## Abstract

Inpatient withdrawal treatment (detoxification) is common in opioid dependence, although dropout against medical advice often limits its outcome. This study aimed to assess baseline predictors of dropout from inpatient opioid detoxification with buprenorphine, including age, gender, current substance use, and type of postdetoxification planning. A retrospective hospital chart review was carried out for inpatient standard opioid detoxifications using buprenorphine taper, in a detoxification ward in Malmö, Sweden (*N* = 122). Thirty-four percent of patients (*n* = 42) dropped out against medical advice. In multivariate logistic regression, dropout was significantly associated with younger age (OR 0.93 [0.89–0.97]) and negatively predicted by inpatient postdetoxification plan (OR 0.41 [0.18–0.94]), thus favouring an inpatient plan as opposed to outpatient treatment while residing at home. Dropout was unrelated to baseline urine toxicology. In opioid detoxification, patients may benefit from a higher degree of postdetoxification planning, including transition to residential treatment, in order to increase the likelihood of a successful detoxification and treatment entry. Young opioid-dependent patients may need particular attention in the planning of detoxification.

## 1. Introduction

Withdrawal treatment (often referred to as detoxification) is a common treatment practice in heroin-dependent patients attempting to quit heroin use and in order to facilitate entry into psychosocial treatment. Intuitively, retention during inpatient detoxification is likely to be of great value in order to initiate and succeed in subsequent treatment [1]. The premature termination of heroin detoxification is common [2], and high rates of relapse into heroin abuse are seen in patients who fail to enter other treatment after detoxification [3].

Buprenorphine is common as medication in opioid withdrawal and has demonstrated good efficacy in such treatment [4–7]. Typically, withdrawal symptoms occurring during detoxification with buprenorphine have been described to be mild [8]. In addition to a withdrawal medication against specific opioid withdrawal symptoms, a limited amount of previous research has evaluated the role of potential risk factors of dropout from detoxification. The abuse of nonopioid drugs prior to admission, including cocaine, has been suggested as a risk factor for dropout from heroin detoxification [8, 9]. Also, apart from the role of withdrawal medication in the prediction of outcome, early data have suggested that completers of detoxification may have a more severe psychological profile, expressed as symptoms on the Symptom checklist 90 (SCL-90) measure [2].

A majority of studies comparing different strategies for opioid detoxification have been pharmacological trials comparing different medications [7], and there has been considerably less research assessing other potential predictors of outcome in this area. Previous data—yet unpublished—from our group indicate that the presence of a postdetoxification plan may increase completion of detoxification [8]. The present study aimed to analyse predictors of dropout during inpatient opioid detoxification treatment, with a focus on baseline urine toxicology and the type of postdetoxification planning, in a setting where the medical management was intended to follow the same principles for all patients, thus, attempting to keep medication a constant factor.

## 2. Method

The present study is a retrospective chart review, using hospital records for pharmacological opioid detoxifications carried out in an inpatient detoxification unit of Malmö Addiction Center, Malmö, Sweden. Here, detoxification refers to a short-term inpatient procedure aiming at detoxifying the patient from her/his primary opioid of abuse (typically heroin, see below), through a procedure where withdrawal symptoms are treated with an opioid agonist which is tapered while the patient remains in the inpatient hospital setting. The setting of the present study is a closed ward designed specifically for the voluntary detoxification of patients with illicit substance use disorders, mostly heroin users. The planning of admissions to this ward always involved a postdetoxification plan for either inpatient residential treatment or outpatient treatment. In the present setting, such postdetoxification psychosocial treatment is planned and financed by social authorities after an active application of the client. The type of postdetoxification planned is made by social authorities in collaboration with the clients, attempting to optimize the treatment plan with respect to the individual needs and other relevant conditions of each patient.

Patients admitted for opioid detoxification in the present ward typically have a severe drug use pattern involving a high degree of illicit drug use, separated from patients with a more pronounced prescription drug abuse who are treated in another part of the treatment organization. According to full-year statistics reported from this ward, heroin is the primary drug of abuse in 98% of patients admitted for opioid detoxification (the few remaining patients reporting methadone, buprenorphine, fentanyl, or morphine).

In the treatment of opioid withdrawal symptoms, patients are observed until they develop subjective and objective withdrawal symptoms, and the medication with buprenorphine is initiated once such symptoms appear. Thereafter, the buprenorphine medication is titrated by an experienced nurse until physical withdrawal symptoms are controlled. From that peak dose, the dose is thereafter tapered gradually and in an individualized manner, but typically reducing the dose by 2 mg daily.

The present study included detoxification episodes with a date of admission from October, 2005, until June, 2007. However, for patients with more than one admission during this period of time, only the first admission was included. Patients admitted during this period of time were included if they underwent detoxification and presented with a withdrawal syndrome requiring medication, and who underwent detoxification with buprenorphine. The period assessed was chosen in order to include only detoxifications carried out after a change of policy in the detoxification center, allowing only admissions for detoxification if a structured postdetoxification plan was set upon admission, involving either an inpatient/residential or an outpatient treatment plan, typically set up by social authorities. Identified inpatient treatment episodes were excluded if they did not explicitly involve withdrawal treatment. We identified 122 unique individuals.

For all patients, urine drug screen (UDS) results and type of postdetoxification plan were registered. Postdetoxification has been shown in unpublished data to have an influence on retention in detoxification [8], and here, where all patients had some kind of such postdetoxification plan, treatment plans were categorized depending on whether they involved a treatment associated with an inpatient treatment or an outpatient treatment. Postdetoxification plans were categorized such that inpatient treatment included structured psychosocial treatment carried out in the context of a residential stay, whereas outpatient treatment comprised all outpatient psychosocial treatment and planning basically involving only housing (in addition to outpatient psychosocial treatment or support). Also, in statistical analysis, age and gender were included as potential predictors to control for. UDS was carried out for opiates, cannabis, cocaine, amphetamine, and benzodiazepines. UDS results were available for 119 patients, excluding three individuals who dropped out before urine testing. Results of the baseline UDS were missing in four cases for cannabis, in five cases for benzodiazepines, in eleven cases for amphetamine, and in thirteen cases for cocaine, leaving 104 individuals for whom a complete UDS was available. Peak dose of the withdrawal medication (buprenorphine) was included in the model, but as early dropouts may not have reached their peak dose (or, in two cases, left before receiving any buprenorphine doses at all), peak dose analyses were also carried out when excluding dropouts who remained for only one day, or excluding dropouts who remained for up to two days, respectively. In the present setting, peak doses are typically reached within one or two days in detoxification.

Statistical analysis involved bivariate comparisons of patients who completed the inpatient detoxification episode (completers, patients who underwent full tapering of medication and were discharged according to the plan) and patients who left the ward prematurely against medical advice (dropouts), using chi-square analysis for categorical variables and Student's *t*-test for continuous variables. Variables which were significantly associated (*P* < 0.05) or which tended to be associated with dropout (*P* < 0.10) were entered (simultaneously) in a multivariate logistic regression analysis with dropout status as the dependent variable. As the results of the UDS were available in all but three patients (*n* = 119) and complete for all included substances in 104 patients, a control analysis was carried out, including only these 104 patients. These 104 patients did not differ from clients with fewer drug tests available (15 clients), with respect to age, gender, or number of drugs reported.

All statistical analyses were carried out in SPSS version 21. Given the legislation for ethical approval in Sweden, the present chart review, carried out in the context of pregraduate medical school research, does not require ethical approval.

## 3. Results

Mean age was 34.3 years (standard deviation 9.2 years), with a median age of 33 years (range 19–56 years). Ninety-nine of the patients (81%) were males. Seventy-four subjects (61%) had an inpatient postdetoxification plan. In the 119 patients for whom UDS data were available, 117 (98%) screened positive for opiates. Among available analyses, cannabis was positive in 42% of cases, cocaine in 12%, amphetamine in 20%, and benzodiazepine in 63% of cases.

Forty-two patients (34%) dropped out of detoxification against medical advice, on average after 6.7 days. Completers remained for a mean of 13.3 days. Among dropouts, 11 (26%) dropped out before reaching peak dosing of buprenorphine (two of whom left before receiving their first dose), after a mean of 1.8 days, 13 (31%) left during buprenorphine treatment and after reaching their peak dose (after a mean of 5.3 days), and 18 (43%) after receiving their final dose of buprenorphine (after a mean of 10.8 days). The mean peak dose reached during detoxification or before dropout was 14.1 mg (SD 5.1, median 13 mg, range 0–24 mg). No significant difference was seen in peak dose between completers and noncompleters (*P* = 0.20). This was confirmed in a secondary analysis eliminating patients who dropped out too early to reach a peak dose, including only patients who remained for more than one day (*n* = 119, 14.6 mg for completers and 14.1 mg for noncompleters, *P* = 0.61) or only patients who remained for more than two days (*n* = 112, 14.6 mg versus 15.3 mg for noncompleters, *P* = 0.49).

Two variables at least tended to be associated with dropout in the binary analysis; completers of detoxification were older (36.0 versus 31.0 yrs, *P* = 0.004, *t* = 3.00) and tended to be more likely to have a postdetoxification plan involving inpatient/residential treatment (*P* = 0.08, *χ* ^2^ = 3.05) rather than an outpatient postdetoxification plan (see Table 1).

In bivariate analysis of all clients who provided a UDS, none of the nonopioid substances assessed at baseline were significantly associated with dropout (although a tendency was seen for cocaine to predict dropout, *P* = 0.09, although this was also the substance with the highest number of missing data). The total number of nonopioid substances in the urines also did not differ between dropouts and completers (*P* = 0.25), and nor did the percentage of patients who screened positive for more than one drug (*P* = 0.74, Table 2). When restricting the analysis to patients for whom no urine drug results were missing, there also was no association between cocaine use and dropout (*P* = 0.30), such that no drug screen variable was further entered in the analysis.

When entering age and postdetoxification plan (the two variables demonstrating at least a trend to predict dropout in binary analyses) in a multivariate logistic regression analysis, dropout was significantly and negatively associated with an inpatient postdetoxification plan (OR 0.41 [0.18–0.94], *P* = 0.03) and with older age (OR 0.93 [0.89–0.97]), thus indicating that older patients and patients with an inpatient postdetoxification plan were more likely to complete detoxification.

## 4. Discussion

The present study aimed to assess predictors of dropout against medical advice during inpatient opioid detoxification and demonstrated that younger patients and patients with only an outpatient postdetoxification plan were at significantly higher risk of dropout. Despite some previous findings indicating that nonopioid drug use may increase the risk of dropping out, no association was seen with urine toxicology results in the present study. The aim was to assess other factors of potential importance, including postdetoxification planning, polydrug use pattern, and demographic data, and the intention was to keep pharmacological strategy as constant as possible, given the principles applied in the present detoxification unit during this period. For this reason, we studied a period of time when buprenorphine taper was the standard procedure in opioid detoxification in this ward, and where dosing procedures were similar, although flexible based on levels of symptoms. Although dosing is difficult to compare for a group where a significant proportion of patients drop out too early from treatment, bivariate data (including data excluding the earliest dropouts) indicated that the groups were comparable regarding peak doses of buprenorphine.

The results of the study may have clinical implications. Short-term detoxification procedures, typically an opioid taper, aim to decrease withdrawal symptoms and facilitate subsequent treatment and abstinence. Despite the obvious role of opioid maintenance treatment with methadone, buprenorphine, or buprenorphine-naloxone [10] in the treatment of opioid dependence, inpatient withdrawal treatment remains, either as a necessity in the acute treatment of withdrawal in patients who discontinue opioid use for various reasons, or in order to initiate any kind of nonopioid treatment such as antagonist treatment or psychosocial treatment. Given the relative paucity of research in this area, our study provides data which call for further research, potentially assessing a wider range of variables possibly predicting the course and outcome of detoxification. In the present study, the type of postdetoxification planning was associated with dropout, indicating that patients with a structured inpatient postdetoxification plan, typically referred to as residential or institution treatment, were more likely to complete detoxification. This may have several explanations. The transfer into institution treatment may require a higher degree of commitment than if a patient's plan is to return home after detoxification and—from her/his home—to attend treatment in an outpatient setting without the total around-the-clock separation from her/his habitual environment. As such, it cannot be excluded that patients who appear to have a higher level of functioning and to be more likely to complete detoxification are offered a higher degree of postdetoxification planning. However, although this study was able to include only a low number of variables, nothing indicated a clear difference between the groups in terms of clinical severity; instead, urine toxicology indicating a common pattern of polydrug use did not reveal any differences likely to indicate a difference in severity between completers and noncompleters. Thus, although results have to be interpreted with caution, one plausible theory is that patients undergoing inpatient detoxification from opioids may perceive a stronger motivation for continued treatment if this involves the direct transfer from an inpatient hospital ward directly into an inpatient residential treatment. As an outpatient postdetoxification plan means that the patient simply returns home upon discharge, patients with a fluctuating level of treatment motivation may be more prone to leave prematurely against medical advice if their discharge plan was to return home, compared to a structured transition into residential treatment. This may be further emphasized by previous data showing that opioid-dependent patients are likely to return to illicit drug use after a short-term opioid taper [11], again causing a greater challenge to patients with only an outpatient followup after detoxification. This topic needs to be elaborated in future research, possibly also in a prospective study design.

Older age was a significant predictor of retention in detoxification, indicating that younger patients were more likely to drop out against medical advice. Further research is needed in order to elaborate on this finding, but the relatively pronounced difference in mean age between completers and noncompleters (five years) indicates that age plays a role in the prediction of retention in this context, or—theoretically—that the effect may be due to a difference in duration of drug use, a variable which could not be controlled for here. Younger patients may not have developed the same degree of negative consequences due to substance use, but this or other possible explanations may need to be assessed in further research.

The present study failed to demonstrate any association between polydrug use or any other specific nonopioid substance and the risk of dropout. While polydrug use in opioid-dependent patients has been shown to complicate opioid use in many aspects, including lower long-term treatment retention and increased overdose mortality [12, 13], there also have been data demonstrating an increased risk of dropout against medical advice from detoxification [9, 14]. This discrepancy may call for further research in the area, as a difference in risk of dropout in different groups of drug users may require differentiated management based on such knowledge.

The present study has limitations. First, the dataset is limited to 122 individuals, and the number of potential predictors included in the study was limited. For example, the presence of other psychiatric disorders than opioid dependence could not be included in the analysis, and systematic evaluation for psychiatric disorders likely would have been strongly biased in these individuals quitting a heavy use of illicit drugs immediately prior to admission. Also, available data on patients' substance use pattern were based only on baseline urine toxicology upon admission, thus, aiming more at describing the most recent drug use than to describe a more stable drug use pattern, and without systematic reporting of actual substance use disorders related to other substances than opioids. In addition, information on the quantity and frequency of substance use prior to admission was not systematically available. In addition, the retrospective nature of the study does not allow for a systematic description of withdrawal severity, such as with an objective symptom screening. Instead, medication has been individualized based on clinical assessment of withdrawal symptoms and should intuitively correlate to individual withdrawal severity, although the present study does not provide any measure to calculate this. Also, as this is a retrospective study with a naturalistic rather than experimental approach, it cannot be excluded that the type of postdetoxification plan is influenced by external factors which, in turn, may influence dropout rates. This risk may be reduced by the control for factors such as age, gender, and the dose required for withdrawal symptom relief, but the potential influence of further factors cannot be excluded. One another limitation is the missing UDS data. For cocaine, missing in 13 cases and the substance most commonly missing in drug tests, we had no systematic information about the reasons for missing cases, which is the reason for conducting an analysis on only cases with all urine data available.

To conclude, the present data indicate that the type of treatment planned to occur after detoxification may affect the possibility of retaining patients in the detoxification procedure. Also, younger patients admitted for detoxification may be at higher risk of dropping out against medical advice.

## Figures and Tables

**Table 1:** Patients undergoing inpatient opioid detoxification. Characteristics of completers versus dropouts.

	Completers (*n* = 80)	Dropouts (*n* = 42)	*P* value	Chi^2^/*t* value
Female gender	16%	24%	0.31	1.03
Inpatient/residential postdetox. plan	66%	50%	0.08	3.05
Age (yrs)	36.0	31.0	<0.01	3.00
Peak dose, buprenorphine (mg)	14.6	13.3	0.20	1.28

**Table 2:** Results of drug screening upon admission to detoxification. Bivariate comparison of positive screenings results between completers and dropouts, with and without clients with any missing drug screen.

	Completers^1^ (*n* = 80)	Dropouts^1^ (*n* = 39)	*P* value^1^	Completers^2^ (*n* = 71)	Dropouts^2^ (*n* = 33)	*P* value^2^
Cannabis, positive	36 (45%)	14 (36%)	0.40	31 (44%)	12 (36%)	0.48
Cannabis missing	0	1				
Cocaine, positive	6 (8%)	7 (20%)	0.09	6 (8%)	5 (15%)	0.30
Cocaine, missing	7	3				
Amphetamine, positive	12 (15%)	10 (26%)	0.15	11 (16%)	9 (27%)	0.16
Amphetamine, missing	5	3				
Benzodiazepines, positive	48 (60%)	26 (67%)	0.42	41 (58%)	24 (73%)	0.14
Benzodiazepines, missing	1	1				
Number of nonopioid substances in urine	1.44	1.67	0.25	1.24	1.52	0.17
>1 nonopioid substance in urine	31 (39%)	15 (36%)	0.74	27 (38%)	14 (42%)	0.67

^1^All clients with urine drug screen results (*n* = 119).

^2^Clients with no missing urine drug screen data (*n* = 104).
